# Health Care Social Media: Expectations of Users in a Developing Country

**DOI:** 10.2196/med20.2720

**Published:** 2013-08-09

**Authors:** Amrita, Dhrubes Biswas

**Affiliations:** ^1^Indian Institute of Technology (IIT)Rajendra Mishra School of Engineering Entrepreneurship (RMSOEE)KharagpurIndia

**Keywords:** health care, social media, developing economy, user expectations, networking, eHealth, online patient care, online medical advice

## Abstract

**Background:**

Affordability, acceptability, accommodation, availability, and accessibility are the five most important dimensions of access to health services. Seventy two percent of the Indian population lives in semi-urban and rural areas. The strong mismatched ratio of hospitals to patients, rising costs of health care, rapidly changing demographics, increasing population, and heightened demands in pricing for technological health care usage in emerging economies necessitate a unique health delivery solution model using social media. A greater disease burden lies in the health care delivery in developing country like India. This is due to the lack of health care infrastructure in the majority of semi-urban and rural regions. New techniques need to be introduced in these regions to overcome these issues. In the present scenario, people use social media from business, automobiles, arts, book marking, cooking, entertainment, and general networking. Developed and advanced countries like the United States have developed their communication system for many years now. They have already established social media in a number of domains including health care. Similar practice incidences can be used to provide a new dimension to health care in the semi-urban regions of India.

**Objective:**

This paper describes an extended study of a previous empirical study on the expectations of social media users for health care. The paper discusses what the users of social media expect from a health care social media site.

**Methods:**

Multiple regression analysis was used to determine the significance of the affect of four factors (privacy, immediacy, usability, and communication) on the usage of health care social media. Privacy, immediacy, usability, and communication were the independent variables and health care social media was the dependant variable.

**Results:**

There were 103 respondents who used the online questionnaire tool to generate their responses. The results from the multiple regression analysis using SPSS 20 showed that the model is acceptable, with *P*=.011, which is statistically significant on a *P*<.05 level. The observed *F* value (2.082) in ANOVA was less than the given value in the *F* table (2.61), which allowed us to accept the hypothesis that the independent variables influence the dependant variable. The users of social media in India expect that they can best utilize social media through emergency service information. They want to be able to learn the operations of the social media site quickly and expect to know about health camps and insurance collaborations. However, people like to become friends with people with similar interests based on their interests identified.

**Conclusions:**

Health care social media requires intelligent implementation in developing economies. It needs to cater to the expectations of the users. The people in India, especially those in urban and semi-urban regions, are very interested in accepting the system.

## Introduction

### Background

Social media is now a buzzword in the new generation of digital communications. Social networks are networks that link people and machines [1]. The way the world saw Web 1.0 during 1990’s [2] has completely changed by the emergence of social media. Mobile and handheld devices having social networking applications at affordable prices have made people more conversant with social media usage. In the present scenario, people use social media right from business, automobiles, arts, bookmarking, cooking, entertainment, and general networking. It has created a highly collaborative virtual environment where individuals and communities share, and modify user-generated content. This process mostly employs mobile and Web-based technologies [3]. Social media has created a change in the society and is getting deployed in various domains. It is being tested for its effectiveness for different levels including health care. This change is dramatic in health care where the focus has shifted from costly high-tech health care to non-traditional health care using social media.

In developing countries, a number of attempts have been made to reform health care for the underprivileged. However, it is mostly the private sector players [4] who create a difference for the underprivileged. Besides the local players and entrepreneurs, there are other successful efforts in health care delivery for developing telemedicine like Voxiva, IBM health care solutions, De Novo Group, Arvind Eye Care System, Narayana Hrudayalaya, and Pilot Projects by Indian Space Research Organization (ISRO) with Apollo Hospital. Moreover, wide prevalence of mobile usage adds to the flexibility of the health care delivery system in India. Recent reports on mobile usage shows that India constitutes 10% of the total mobile usage in the world. This is very clear when we look at the 1.2 billion population residing in India out of which 72% belong to rural areas.

Moreover, technology has influenced the spread of information and the manner it can be disseminated to the world. Media and its landscape has seen significant transformation in the last decade and social media is increasingly replacing the traditional media [5-8]. The wide acceptance of social media for the last few decades has triggered research comparing traditional media and social media [9]. They have been analyzed for usefulness in various domains such as marketing communication, cost effectiveness, sales performance, health care and so on. An individual's social network is the one surrounded by network of relationships and its ties [10]. A general tendency of influence of social media has been noted from the online users, for instance, book reviews affects the sales [11,12]. However, researchers have also confirmed the interrelatedness of both the media [6,9,13].

The developed and advanced countries like the United States have developed their interrelated communication system many years from now. This includes the usage of social media in almost every domain including health care. Manhattan Research Group found long back in 2002 [14] that the total e-Patients zone of influence was 166.5 million Americans. The introduction of new technologies and interrelated media has made the society well informed about the happenings in the other parts the world too in various ways. The modern Indian society is now well informed of the new technologies being developed for health care too. Conversely, they are ignorant about the learning, usage, and outcomes of the same. There lies the issue of “Technology to Health (T2H)” Gap [15].

The world average of beds per 1000 patients is 2.6 where as in India it is only 0.7 [16]. This is just an example of poor infrastructure in India. Chaudhry et al, (2005) [17] has given a picture of Indian villages which has also revealed that households descent into poverty [18] due to three principal factors, that is, health expenses, high-interest private debt, and social and customary expenses of which health care expenses figured prominently in more than half of all cases of decline into poverty. Countries all around the world especially the BASIC (Brazil, South Africa, India, and China) are struggling to address the ever-increasing costs, poor or inconsistent quality, and inaccessibility to timely health care.

Everyone needs similar levels and quality of health care services particularly five dimensions of access to health services as affordability, acceptability, accommodation, availability, and accessibility [19,20]. The strong mismatch of ratio of hospitals to patients, rising costs of health care, rapidly changing demographics, increasing population and heightened demands in pricing for technological health care usage in emerging economies necessitate a unique health care reform. The health care system is getting unmanaged by the high-technology introduction as well as high price points of the interest groups. Furthermore, it is adding to the already existing realm of new and costly technologies in health care [21].

However, these challenges can be relieved for those who might use information technology to an extent by knowing about similar kinds of patients with same disease patterns, share their experiences and many more by the introduction of a one step ahead social media tool for health care. Thus, social media for health care as technology intervention strategy in information technology may exert their influence through both volume and price effects. Technological interventions at every stage in innovation will direct to sustainable health care system especially in the emerging economies context. Research has also confirmed the value addition and trust involved in a continuous online development of the contents for patients [22].

The augmentation of health care delivery system needs a large reform in the developing economy context. This is directly derived from the poor health care scenario presented in the semi-urban and rural regions. The reform through information and communication tools (ICT), that is, social media might be looked at provided the users are given training. This leads to various research issues. They are: What are the factors that determine the health care social media? Would the people in semi-urban and rural regions of developing countries prefer the intervention to other existing systems? How much information sharing would they be comfortable with? How much would they expect from the health care social media given to them? Hence, this leads to an organized and methodical study of these issues.

This paper makes an attempt to analyze the expectations for health care social media of the already existing users of the social media. The expectations are measured in terms of Privacy, Immediacy, Usability [23], and Communication [24,25].

### Technology to Health Gap in India

It is true to state that these health care reforms are seen mostly in the developed countries. There are few instances of social media usage for health care in the metropolitan areas in India. The world average comparison is just an example of poor infrastructure in India. Amrita et al 2010, [15] mentions that the “T2H Gap” in health care originates from the adversities of affordability, accessibility, and availability. An adoption of proper ICT based health care model for cheaper and quality health care can alone solve this problem to a great extent.

It has also been observed that the wide prevalence of mobile usage adds to the flexibility of the health care delivery system in India. Recent reports on mobile usage shows that India constitutes 10% of the total mobile usage in the world. The Internet users in India are 11.4% [26], which shows information technology can provide a new platform for them.

The World Health Statistics 2012 [27] shows the inadequate health care infrastructure and workforce. The urban regions have four times more doctors and three times nurses than in the rural regions in India. Even though India has quality education and medical institutes, most of them are located in urban regions. This results in health care services deficits in semi-urban and rural regions. The private health sector is currently leading in the service delivery. The statistics show it has 80% of all doctors, 26% of nurses, 49% of beds, and 78% of ambulatory services and 60% of in-patient care. This leads to maximum out-of-pocket expenditure by the large semi-urban and rural population. The gap in health care demand and supply can be met when we implement unique service delivery models in health care using social media.

The motivation of this paper lies in the huge prevalence and acceptance of viral marketing and social media marketing by the people in India. It shows that they are open to new dimensions for comfortable lives. It has already been seen that the drug companies are using social-media to promote their brands. Research has reported positive inclination towards the belief levels for using wiki-based information for health care [28]. Hence, information technology through social media can be used to create better health care information in India.

### Why Health Care Social Media in India?

There are a number of health care networks which includes doctors, patients, nurses, pharmacists and who so ever are interested in health care. There are also a number of applications in the Internet including Google health, medicine 2.0 and health 2.0. All of them target to the population who are already using Internet and can understand computer and information technology. It has been seen from the survey of Internet, that there are many social media networks which deal with doctor networks, nurses’ networks, popular disease support forums, health blogs, patients’ voices, and expert answers.

It might also be stated that various efforts have been made to make health care accessible for the rural and semi-urban population. These efforts are more towards the use of mobile and hand held devices for transferring patients’ information to the relevant doctors. Mobile hospitals and similar efforts have also been made in parts of Africa and Brazil.

The growing body of literature on social media and health care is generally concerned with the advertisers to find new customers. However, scarce literatures of social media for effectiveness of health care especially in developing countries have been viewed.

Mobile phones for health care are on the cusp of spurring an information revolution in such regions [29]. Studying the expectations and influencing variables of a social media network around the usage of mobile technology in coordination of a public-private-partnership might lead to the cost cutting of expenditure on health care. Lot of work is being done in social media domain for finding effectiveness of marketing, advertising and consumer relations. However, it has not been seen in the perspective of health care delivery in semi-urban setting in emerging economy. Authors present in this paper the expectations of the users of developing economy, who would intend to use such a health care social media. These expectations in future can be utilized to implement such system for the semi-urban and rural regions in such economies.

### Objective

Social media provides an substantial amount of information, having the potential to attract significant audience [30]. The similar practice incidences can be used to provide a new dimension to the health care in Indian semi-urban regions. Before we move to the specifications of understanding what the semi-urban users of social media in India, it is required that we understand what the existing users expect. The factors that affect the users of health care social media were found in the previous work [24]. These factors are not tested to answer the key question: Which are the most influencing variables that affect the expectations of users of health care social media in a developing country?

There are a number of health care networks, which includes doctors, patients, nurses, and pharmacists, who are interested in health care. Most of them target to developed and advanced countries. There are not many studies that refer to the developing country perspective. Hence, the objective of the paper is aligned with the aim to understand the social media users in developing country. This might lead us the way to realize how we could proceed further for building any social media tool for developing countries.

## Methods

### Overview

Researchers on social media techniques have mentioned seven functional elements [3]: identity, conversations, sharing, presence, relationships, reputation, and groups. Many other researchers have confirmed and cited these building blocks of social media as attributes for online strategy, public affairs [31], product development through co-creation, tourism, health care [32], and many more not mentioned here.

This study is based on the primary data collected during January 2013 using a survey questionnaire form created in the Google forms in the Internet. This work is based on our previous published paper [24], where we determined significant components for health care social media. We reported in our previous work that: privacy, immediacy, and usability are most the significant factors for health care social media. The results showed that the users of social media have significant privacy value for their health care issues on the Internet. At the same time face-to-face meeting is rejected. The opinions only from doctors would have negative influence, at the same time also not being open for frank suggestions. However there is a preference to be viewed as part of interest columns on health issues. Simple user interface has a larger acceptance than the advanced user settings. Learning from the earlier work and having unstructured discussions with few of the respondents, we added one more factor, that is, communication for further investigation in this work.

The extensive use of social media has already perturbed the common understanding of the *privacy*, [3] though the privacy ideology remains the same as earlier. The users understand that the organization of information in such a way which maintains the individual decorum and independence. The immediacy and communication [3] are the vital parts for sharing information on health care interests. In the presence of above components, another important issue is *usability* preferences, which would determine how frequently the users like to visit the health care social media.

Hence, learning from the previous work and results, we designed the factors to: *privacy, immediacy, usability,* and *communication* in the current work. The first three items were confirmed from previous work. The last item emerged from the learning with unstructured interviews of more than 46 users.

### Sampling Method

The paper is based on the premise that the health customer is able to choose from where and whom they get treated or prefer some close relatives advices for taking such decisions. The users are free to use and have their views on health information over the Internet or social media.

Since we have targeted the users of the social media as the target group, we did not define any premise of distance and place of stay. The only clause we have used for the respondents is that they should be Indian citizens staying in India. Hence we have relied on the snowball sampling method to spread the online survey link. This also helped us identify the few duplications and quality of information.

### Questionnaire Design

Based on our previous experience of the published paper, we designed our questionnaire to remove as well as include the defining constructs for 4 identified factors. Additionally, we designed questions to know the social media presence, preference and health care social media. The distribution of the questions were as such that 42 items were created, 5 each for 4 independent variables and 4, 7, and 11 for social media presence, health care preference, and demography.

The questionnaire was designed as a webpage form using the Google forms available online. The link was shared online through emails, Facebook, Twitter, and interest forums. Sections A to D were designed using 5 point Likert scale. Options ranging from “Strongly Disagree” to “Strongly Agree” for A to C and “Never” to “Always” in the case of D was used. Sections from E to G were majorly multiple choices along with other few to enter themselves as well as select from given choices.

### Data Collection

The response of the online Google form automatically got registered in the Excel format. The response rate was good during the first time intimation and dropped after a few days. After 5 to 6 reminders, 103 responses were generated. It is assumed that the sample is random attributing to the wide demography of the respondents. The data points count, that is, n=103 we can say referring to the Central Limit Theorem (n>30) that the sample size is large and normally distributed.

The idea of using online data collection was generated for the reason that our focus was more on the users of the social media and Internet. Moreover, the large audience, reduced cost of travel, quick time to gather responses, easy data management, and less item non-response led to the decision of online data collection.

The demographic profile of the respondents’ show that majority are between the age groups of 19 to 25. Qualification is majorly in graduation and post graduation. Occupation-wise most of them are professionals. Maximum belong to urban and semi-urban regions. The income shows that maximum have the average income between 1819 to 7273 USD but the next income group has more than 14,545 USD. The sample is representative of the social media users keeping in mind the domicile status. Conversely, maximum response is from the age group 25 to 30 years and below. This shows that the online social media users in India are mainly the younger generation.

## Results

### Variables

The four determinants—privacy, immediacy, usability, and communication of health care social media—have been taken as the predictor variables pertaining to multiple regressions. Health care social media has been considered as the dependent (outcome) variable.

### Hypothesis

Our null hypothesis for determination of the regression has been taken such that the four independent variables (privacy, immediacy, usability, and communication) do not depend on the dependent variable health care social media. Hence, the null hypothesis was designed as health care social media is not dependent on privacy, immediacy, usability, and communication, and are not related. Therefore, the alternative hypothesis is that health care social media is dependent on the variables privacy, immediacy, usability, and communication. Based on the null hypothesis, several propositions are drawn to form the conceptual model (Textbox 1).

The data obtained from survey was regressed using the SPSS 20 package for analysis. We present the results of the regression from Model fit statistics in Table 1.

Table 1 depicts the variability of the data through *R* ^*2*^. The value of .337 shows that a fair amount of variability lies in the dataset. However, the adjusted *R* ^*2*^ show a lesser amount of variability.

The significance value in ANOVA (Table 2, at 90% confidence interval) shows .011, which is less than 0.05. Hence the model is considered as significant. As per Table 1 results, we obtained the observed value of the *F*_20,82_=2.082. However the test statistics from the *F* distribution table for *F*_20,82_=2.61, which is greater than the observed value. Hence this rejects the null hypothesis.

So it can be said that the independent variables privacy, immediacy, usability, and communication has an influence on the dependent variable, health care social media. Accepting the alternate hypothesis, we proceed to explain the significant influences of the independent variable through the reporting of unstandardized coefficients (Table 3).

**Textbox 1: Propositions of the conceptual model.**

Proposition H1: the predictor variable “privacy” has no influence on health care social media.

Proposition H2: the predictor variable “immediacy” has no influence on health care social media.

Proposition H3: the predictor variable “usability” has no influence on health care social media.

Proposition H4: the predictor variable “communication” has no influence on health care social media.

**Table 1:** Reporting of the model summary.^a^

Model	*R*	*R* ^*2*^	Adjusted *R* ^*2*^	Standard error of the estimate
Statistics	.580^b^	0.337	0.175	1.104

^a^Dependent variable: Health care social media

^b^Predictors: (Constant), Communication 5, Communication 4, Privacy 1, Communication 2, Immediacy 1, Privacy 4, Usability 5, Immediacy 3, Usability 2, Immediacy 5, Communication 1, Communication 3, Privacy 5, Privacy 2, Usability 3, Immediacy 2, Usability 4, Privacy 3, Usability 1, Immediacy 4

**Table 2:** Reporting of ANOVA^a^ statistics.

Model	Sum of squares	Degrees of freedom	Mean square	*F*	Significant difference
Regression	50.743	20	2.537	2.082	.011^b^
Residual	99.936	82	1.219	N/A	N/A
Total	150.68	102	N/A	N/A	N/A

^a^Dependent variable: Health care social media

^b^Predictors: (Constant), Communication 5, Communication 4, Privacy 1, Communication 2, Immediacy 1, Privacy 4, Usability 5, Immediacy 3, Usability 2, Immediacy 5, Communication 1, Communication 3, Privacy 5, Privacy 2, Usability 3, Immediacy 2, Usability 4, Privacy 3, Usability 1, Immediacy 4

**Table 3:** Reporting of coefficients.^a^

Model		Unstandardized coefficients		Standardized coefficients	*t*	Significant difference
		Beta	Standard error	Beta		
Constant		2.86	1.19		2.404	0.018
**Privacy**						
	1	-0.009	0.1	-0.011	-0.093	0.926
	2	0.001	0.114	0.001	0.005	0.996
	3	-0.182	0.158	-0.14	-1.152	0.253
	4	0.044	0.145	0.039	0.308	0.759
	5	-0.222	0.126	-0.19	-1.76	0.082
**Immediacy**						
	1	-0.104	0.102	-0.103	-1.023	0.309
	2	0.154	0.117	0.148	1.322	0.19
	3	0.007	0.127	0.006	0.054	0.957
	4	-0.298	0.204	-0.202	-1.463	0.147
	5	0.015	0.241	0.009	0.06	0.952
**Usability**						
	1	0.457	0.184	0.316	2.48	0.015
	2	0.003	0.104	0.003	0.03	0.976
	3	-0.087	0.177	-0.059	-0.495	0.622
	4	0.017	0.152	0.013	0.109	0.913
	5	0.026	0.106	0.026	0.244	0.808
**Communication**						
	1	0.116	0.132	0.094	0.883	0.38
	2	-0.16	0.103	-0.153	-1.55	0.125
	3	0.075	0.117	0.067	0.646	0.52
	4	0.354	0.095	0.405	3.732	0
	5	0.019	0.1	0.019	0.186	0.853

^a^Dependent variable: Health care social media

Looking at the smaller significance level of the model items in Table 3, we can see that privacy 5, usability 1, and communication 4 are highly influencing the dependant variable. We would also like to report the observation based on good difference between *t* value and significance value. The model items in underline are the ones we are interested into. Privacy 5 was intended to find the face-to-face meeting expectations of the users of social media. It has a negative influence on the dependant variable. Usability 1 was intended to know how quickly the users expect to learn a new health care social media launched. It has positive influence. Communication 4 was intended to learn from the users of how they would utilize a health care social media during the situation of emergency. This has very high significance level and positively influences the dependant variable.

Now considering the observations based on the good difference between *t* and significance level, we estimate the following points. Privacy 2 has a fair influence stating that the users expect to become friends with chain system of referrals through friends. Privacy 3 has considerable negative influence on the health care social media. This reveals that the users expect that they should not have control on the disclosure of the health care interests. Though the variable immediacy did not show a very high significance level, all the expectation constructs can be considered. Hence it shows somewhat influence on the dependent variable. Immediacy 1 and 4 has negative influence stating that the users expect not to get advices only from doctors and have cost comparisons of hospitals. Immediacy 2, 3, and 5 have positive influence showing the expectations of users to get opinions from experienced people, insurance companies collaboration with hospitals information and information about free health camps. Usability 2 shows a positive influence stating that they expect to get training from someone to learn the health care social media. Usability 3 has a negative influence that the users do not want to keep administering their settings. Communication 2 has a negative influence on the users where they expect that the physician-patient interaction is not very necessary to join each other in health care social media.

## Discussion

### Principal Findings

The implication of usefulness of social media has been well understood through its usage in marketing and other dominant domains. Social media has seen a good influence in the behaviors of the users in developing economies.

This paper is a contribution of how the users expect and understand the health care social media in India as a developing country. The majority of responses from urban and semi-urban domicile population show that they expect that health care becomes more accessible and available. We show how we can refer to the gap of understanding the impact of how the social media can help semi-urban and rural population in health care. The results would help the designers of health care social media to understand the expectations of the semi-urban and urban population in a developing economy. The results show that people would use the social media sites for health. However there is a need of good awareness and training for making it a successful implementation.

### Limitations

The paper has used snowball sampling for online data collection. This method does not report the response rate of the survey. Even though the different online ways were used to distribute the survey link, getting a large population sample remained a problem same as in traditional data collection. Moreover, we do not know the conditions and setting of the respondents at the time of taking the survey.

The bias of the volunteer sample in the earlier work [24], led us to the introduction of a new independent variable, that is, communication, in the current work. Hence, the online data collection sometimes leads to the possibility of introducing new ideas and factors. This sometimes biases the selected sample. Furthermore, this sample might not include those respondents who use the social media sites less frequently due to the lack of good Internet availability. It is also worth mentioning that this type of survey might have some demographic related biases, such as younger people filling in the online survey.

### Comparison With Prior Work

The current work shares the similar model of regression as the previous one for measuring the expectations of the users of health care social media. The users in the developing economy are conscious about the openness of the privacy in a public forum. There is a variation from the previous work in which we have tried to understand the communication influences between the users. The negative influence of communication for face-to-face patient-physician interaction shows that people are skeptic towards revealing their identity. This is again confirmed by the negative influence results of controlling identity settings. Both the work shows a positive influence of usability for simple and quick learning health care social media.

### Conclusions

The openness of the privacy component was highlighted where it shows negative influence. Users are very skeptic towards keeping their identity and friend’s list open. The less disclosure of health care interests is very prominent. Hence openness of privacy negatively influences the dependant variable. Respondents wish to get advices from experienced people and not only from health experts. Hence immediacy has a positive influence in terms of intermediary communications supported. Usability shows a positive influence where people want to be in directory listings. Communication has a strong positive influence where the users want emergency information over the health care social media.

## Multimedia Appendix 1

Questionnaire.

## Abbreviations

BASIC: Brazil, South Africa, India, and China
ICT: information and communication tools
ISRO: Indian Space Research Organization
T2H: Technology to Health
