# Supplementary material for: Health Care Social Media: Expectations of Users in a Developing Country
Source: Med 2 0. 2013 Aug 9;2(2):e4. doi: 10.2196/med20.2720 (PMC4085124; doi:10.2196/med20.2720)
Supplement: Supplementary file 1 [file med20_v2i2e4_app1.pdf]

Questionnaire

Dear Respondent,

Please take your time to fill up the following questionnaire, as it will help us to design a highly informative and effective platform for the concerned people who are in need of high quality healthcare services at a reasonable price. The questionnaire is divided into sections related to your social media preferences for healthcare, about your usability preferences and demographic details. Thanks for your time and support.

Sincerely,
Amrita,
Research Scholar IIT Kharagpur

	Strongly Disagree	Disagree	Neutral	Agree	Strongly Agree
A. Your Privacy Issues Concerned with the Social Media Network for healthcare.<br>1. Social media network in healthcare should allow users to see everybody`s profile.<br>2. Users can send request to friends of friends and their friends<br>3. Users should have full control on disclosing their identity.					
4. Users should have control on their disclosure of healthcare interests.					
5. You would like to meet your friends of health groups in person.					
B. **Your preference based on the requirements of how much intermediary communication for healthcare social media is comfortable.**					
1. You would like to get suggestions only from doctors.					
2. You would like to get suggestions only from people with some experiences.					
3. You would be happy to get information about Insurance					

company collaboration with the hospitals.					
4. You would be happy to see cost comparisons of various hospitals.					
5. You would be happy to get the information about free health camps.					
C. **Your preference based on the usability based on user friendliness of the healthcare social media.**					
1. You would like to learn how to use the site in just few hours.					
2. You would like to get help from someone to learn the operations of the site.					
3. You would like to administer your setting and privacy all the time.					
4. You would like to be part of directory listings in the interests column.					
5. You prefer telephonic conversations over social network applications for discussion.					
	Never	Seldom	Don't know	Occasion ally	Always
D. **Communication**					
1. You would be confident to follow the suggestions through videos/blogs from people.					
2. You believe that Physician-patient interaction is important before joining together in social media.					
3. You believe that Social networking sites have potential for improving doctor patient communications.					
4. You prefer to use social networking site in case of					

medical emergency (eg. Blood requirement, best doctor, surgery done, etc.)					
5. You prefer other media (TV, newspaper, radio) over social media for medical information.					
E. **About your social media presence**					
1. Do you have separate email ids for	Official	Personal	Friends	Business	One email account for all purpose
2. The following are list of top social media websites. Please mark which ones of the following you use:	o Facebook<br>o Twitter<br>o LinkedIn<br>o MySpace<br>o Ning<br>o Google Plus+<br>o Tagged<br>o Orkut<br>o hi5<br>o myyearbook<br>o Meetup<br>o Badoo<br>o bebo<br>o mylife<br>o friendster<br>o I do not use any social site<br>o Other:				
3. How do you rank the above social networking sites? Please check first 5 you think are good.	o Facebook<br>o Twitter<br>o LinkedIn<br>o MySpace<br>o Ning<br>o Google Plus+<br>o Tagged<br>o Orkut<br>o hi5<br>o myyearbook<br>o Meetup<br>o Badoo<br>o bebo<br>o mylife<br>o friendster<br>o I do not use any social site<br>o Other:				

4. What do you like most in the above highest rated social sites?	o User interface<br>o Privacy Settings<br>o Themes<br>o Friends List view<br>o Create groups option<br>o Photos sharing<br>o Wall Posts<br>o Videos sharing<br>o Friends' Update<br>o Game requests<br>o Application requests<br>o Advertisements in the side<br>o Like style<br>o I do not use any social site.				
F. **Healthcare Related Observations**	Never	Seldom	Don't know	Occasion ally	Always
1. You will prefer to use a complete all-in-all social networking site for HEALTHCARE.					
2. You are familiar and have used health and medical social media sites.					
3. Please check those which you know from the following health and medical social media sites.	o Health 2.0<br>o Medecine 3.0.com<br>o Healthranker.com<br>o OrganizedWisdom.com<br>o PeoplesMD.com<br>o Trusera.com<br>o American Well.com<br>o Daily Strength.com<br>o Group loop.com<br>o Mamaherb.com<br>o MDjunction.com<br>o Twit2fit.com<br>o Vitals.com<br>o PatientsLikeMe.com<br>o RealSelf.com<br>o Never searched any health site<br>o Other:				
4. You try to search a feedback about the hospital/doctor before					

visiting.					
5. You try to search health related information from…	o Internet<br>o close relatives<br>o close friends even if they are residing in far away places<br>o Other:				
6. You recommend your friends to search about any health information from the internet.					
7. You have used medical applications in mobile phones.					
G. **Demographic Information**	**Full Name**				
	**E- Mail:**				
	**Birth Year:**				
	**Gender**	Male		Female	
	**Qualification**	o Matriculation<br>o Higher Secondary<br>o Graduation<br>o Post-Graduate<br>o Professional<br>o None of the Above			
	**Occupation**				
	**No. of Members in the family**				
	**No. of earning members in the family**				
	**Family Annual Income (Rs):**	o Less than 50,000<br>o 50,000 to 1 Lakh<br>o 1 lakh to 4 lakhs<br>o 4 Lakhs to 8 lakhs<br>o 8 lakhs or more			

**Place of Domicile**	Rural	Semi-Urban	Urban	Metropolitian
**Nationality**				
